# Proton-Assisted Amino Acid Transporter PAT1 Complexes with Rag GTPases and Activates TORC1 on Late Endosomal and Lysosomal Membranes

**DOI:** 10.1371/journal.pone.0036616

**Published:** 2012-05-04

**Authors:** Margrét H. Ögmundsdóttir, Sabine Heublein, Shubana Kazi, Bruno Reynolds, Shivanthy M. Visvalingam, Michael K. Shaw, Deborah C. I. Goberdhan

**Affiliations:** 1 Department of Physiology, Anatomy and Genetics, University of Oxford, Oxford, United Kingdom; 2 Sir William Dunn School of Pathology, University of Oxford, Oxford, United Kingdom; New Mexico State University, United States of America

## Abstract

Mammalian Target of Rapamycin Complex 1 (mTORC1) is activated by growth factor-regulated phosphoinositide 3-kinase (PI3K)/Akt/Rheb signalling and extracellular amino acids (AAs) to promote growth and proliferation. These AAs induce translocation of mTOR to late endosomes and lysosomes (LELs), subsequent activation via mechanisms involving the presence of intralumenal AAs, and interaction between mTORC1 and a multiprotein assembly containing Rag GTPases and the heterotrimeric Ragulator complex. However, the mechanisms by which AAs control these different aspects of mTORC1 activation are not well understood. We have recently shown that intracellular Proton-assisted Amino acid Transporter 1 (PAT1)/SLC36A1 is an essential mediator of AA-dependent mTORC1 activation. Here we demonstrate in Human Embryonic Kidney (HEK-293) cells that PAT1 is primarily located on LELs, physically interacts with the Rag GTPases and is required for normal AA-dependent mTOR relocalisation. We also use the powerful i*n vivo* genetic methodologies available in *Drosophila* to investigate the regulation of the PAT1/Rag/Ragulator complex. We show that GFP-tagged PATs reside at both the cell surface and LELs *in vivo*, mirroring PAT1 distribution in several normal mammalian cell types. Elevated PI3K/Akt/Rheb signalling increases intracellular levels of PATs and synergistically enhances PAT-induced growth via a mechanism requiring endocytosis. In light of the recent identification of the vacuolar H^+^-ATPase as another Rag-interacting component, we propose a model in which PATs function as part of an AA-sensing engine that drives mTORC1 activation from LEL compartments.

## Introduction

Mammalian Target of Rapamycin, (mTOR), is a critical integrator of nutrient, energy and growth factor signals in higher eukaryotes [1]. This kinase controls several key cell biological processes, including protein translation, growth, the cell cycle and autophagy. Defective mTOR signalling has been linked to a range of major human diseases, including cancer [2], obesity [3], Type 2 diabetes [4], [5] and several neurodegenerative disorders [6], as well as having evolutionarily conserved effects on ageing [7], [8].

Modulating mTOR activity could, therefore, have important therapeutic implications for the treatment of human disease and promoting healthy ageing. Indeed, strategies involving analogues of the mTOR inhibitor rapamycin, so-called ‘rapalogs’, have been approved by the FDA for the treatment of patients with advanced renal carcinoma [9]–[11]. However, mTOR exists in at least two characterised multicomponent complexes, mTORC1 and mTORC2. Rapamycin and the rapalogs have been shown to have the strongest effect on mTORC1, but there is some evidence that they can also inhibit mTORC2 [12]. mTORC1 negatively feeds back on Akt, a key target of growth factor signalling that promotes mTORC1 activity. Furthermore, mTORC2 positively regulates Akt [13]. There has therefore been recent interest in developing drugs that target both mTORC1 and mTORC2, such as the ATP-competitive mTOR kinase inhibitors [14], [15]. However, the mTORC1 signalling pathway is globally active in all cells, so directly targeting mTOR is likely to have significant side effects. A preferable strategy might be to focus on any nutrient-sensing mechanisms employed selectively by cancer cells that allow them to compete successfully with their normal neighbours [16].

The growth factor-regulated phosphoinositide 3-kinase (PI3K)/Akt pathway is antagonised by the major human tumour suppressor gene, *PTEN*, and is known to be hyperactivated in the majority of human cancers [17], [18]. Genetic analysis in flies has been particularly helpful in establishing a link between PI3K/Akt and the TORC1 signalling cascade via the G protein Rheb and its antagonist, the Tuberous Sclerosis Complex (TSC; [19], [20]). There is extensive experimental evidence in flies [19], [21], [22], mammalian cell culture [23] and mouse models [24] that in a nutrient- and growth factor-depleted microenvironment, in which for example, tumour cells often grow preferentially [25], increased PI3K/Akt and mTORC1 signalling can give cells a major growth advantage.

Despite the central importance of mTORC1 in several basic cellular functions, some aspects of its regulation are still poorly characterised. For example, we do not understand the mechanism by which extracellular amino acid (AA) levels are ‘sensed’ inside cells and how they synergise with PI3K/Akt signalling to stimulate mTORC1 [26], [27]. In a recent breakthrough, the Rag GTPases were shown to specifically modulate TORC1 signalling in cell culture and *in vivo*, using genetic approaches in *Drosophila* [28], [29]. They function as a heterodimeric complex between RagA or RagB and RagC or RagD; which is involved in an AA-dependent process that relocalises mTOR to late endosomes and lysosomes (LELs; [29], [30]), promoting assembly and activation of mTORC1. This aspect of mTOR activation appears to be evolutionarily ancient, because yeast TOR1 localises with the EGO complex, which contains the yeast Rag GTPase orthologues, Gtr1p and Gtr2p, and other regulatory components, such as the guanine nucleotide exchange factor Vam6, near the vacuolar membrane, a lysosome-like structure [31], [32]. A trimeric complex of proteins, dubbed the Ragulator, has been implicated in binding the Rag GTPase heterodimer to the lipid bilayer [30].

However, the AA sensing mechanisms that drive the process of mTOR relocalisation and activation remain unclear [33]. Membrane-associated molecules involved in this mechanism are likely to be good therapeutic targets to combat tumour growth [34], particularly if the regulatory mechanisms change as PI3K/Akt signalling increases and cells become more resistant to alterations in extracellular AA levels. Studies in cell culture have highlighted several cell surface amino acid transporters (AATs), including the solute carrier (SLC)1A5 glutamine transporter and the heterodimeric CD98 (SLC7A5/SLC3A2) bidirectional AA exchanger [35] that mediate the uptake of AAs. There are also a number of intracellular signalling molecules other than the Rag GTPases, e.g., MAP4K3 [36]–[38] and Vps34 [39], [40], which are thought to be involved in mediating the AA-dependent signal to mTORC1 (reviewed in [26]), but there is no evidence that these molecules act directly as AA sensors.

Other studies have shown that once the mTORC1/Rag/Ragulator complex is assembled on LELs, a proton gradient across the LEL membrane is required for mTORC1 to activate its downstream targets [41]. During starvation, when cells enter autophagy and recycle intracellular organelles and macromolecules to promote survival, mTORC1 is reactivated from autolysosomal membranes in response to accumulation of intralumenal AAs [42]. However, the AA sensing mechanism involved here has also not been identified.

In a screen of different AATs for *in vivo* growth effects in *Drosophila*, we found that a specific class of AAT, the Proton-assisted (PAT or SLC36) Amino acid Transporters (reviewed in [43]–[45]), has a particularly potent effect on TORC1-mediated growth. The TORC1-regulatory role of the PATs is conserved in humans [46]. Genetic and biochemical evidence suggests that in response to AAs, the ubiquitously expressed human PATs, PAT1 and PAT4, promote phosphorylation of key downstream targets of mTORC1 and are required for growth. Furthermore, in rapidly growing cells, such as human embryonic kidney (HEK-293) and MCF-7 breast cancer cells, PAT1 is localisation is intracellular [46], suggesting that it is not involved in AA influx into the cell, but in another downstream event in mTORC1 activation.

Here we show that in HEK-293 cells, PAT1 physically interacts with Rag GTPases and co-localises with these molecules on LELs. PAT1-positive LELs recruit mTOR upon exposure to AAs. We also present evidence that PI3K/Akt/Rheb signalling promotes PAT-dependent growth in flies via a mechanism that involves endocytosis of cell surface PATs. Our data therefore suggest that PATs and Rag GTPases complex at the surface of LELs to promote activation of TORC1-mediated growth, and that growth factor-mediated PI3K/Akt/Rheb signalling mediates some of its effects on TORC1 by regulating the accumulation of this complex inside normal cells. We propose a model to explain how the interaction of AAs with PATs might be a critical part of the AA sensing mechanism that drives TORC1 activation.

## Results

### mTOR is recruited to PAT1-containing late endosomes and lysosomes upon AA stimulation

**Figure 1:**
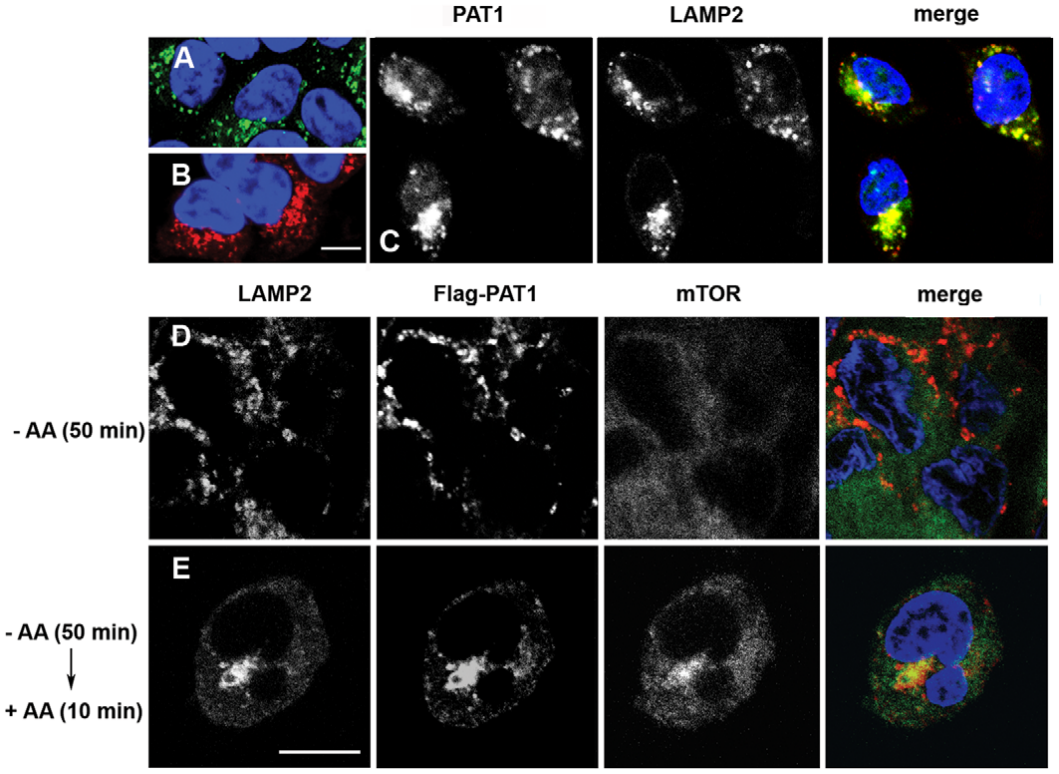
mTOR localises to LAMP2/PAT1-positive compartments upon AA stimulation. (**A**, **B**) Flag-PAT1 (red; B) overexpressed in a stably transfected HEK-293 cell line has a similar intracellular localisation pattern to endogenous PAT1 (green; A). Cells under steady state conditions are shown. (**C**) Endogenous PAT1 strongly co-localises with LAMP2, which marks LEL compartments. Cells were stained after 50 min AA starvation followed by 10 min AA stimulation. Merge shows LAMP2 (red), PAT1 (green) and DAPI (blue). (**D**, **E**) Subcellular localisation of LAMP2, Flag-PAT1 and mTOR after 50 min AA starvation (D) and 50 min AA starvation followed by 10 min AA stimulation (E). Note that Flag-PAT1 and LAMP2 co-localise under both conditions, but mTOR is only recruited to a limited number of LELs upon AA stimulation. Merge shows Flag-PAT1 (red), mTOR (green) and DAPI (blue). Scale bars in B and E are 10 µm, A; scale bar in B also applies to A and C, scale bar in E also applies to D.

We have previously shown that the two ubiquitously expressed PATs, PAT1 and PAT4, are critically required for activation of mTORC1 by extracellular AAs in rapidly growing cultured cells, even though PAT1 is predominantly located within cells [46]. To determine the subcellular compartments in which PAT1 is localised, we generated a stable HEK-293 cell line overexpressing Flag-tagged PAT1, see supplemental methods (Methods S1). Under steady state growth conditions, this fusion protein has a punctate intracellular expression pattern, which is often primarily localised in an asymmetric perinuclear cap (Figure 1B), and resembles the expression of endogenous PAT1 (Figure 1A). Co-labelling either non-transfected HEK-293 cells or Flag-PAT1-expressing cells with an antibody against the LEL marker LAMP2 revealed extensive co-localisation in all cells (greater than 80% co-localisation [n = 20] for both Flag-PAT1 and endogenous PAT1). These molecules co-localised both in AA-starved cells (Flag-PAT1 in Figure 1D) and after cells were first depleted of extracellular AAs, then stimulated with AAs for 10 min (PAT1 in Figure 1C and Flag-PAT1 in Figure 1E). We conclude that PAT1 is primarily located in LEL compartments in HEK-293 cells and that this distribution is not affected by the presence of extracellular AAs.

**Figure 2:**
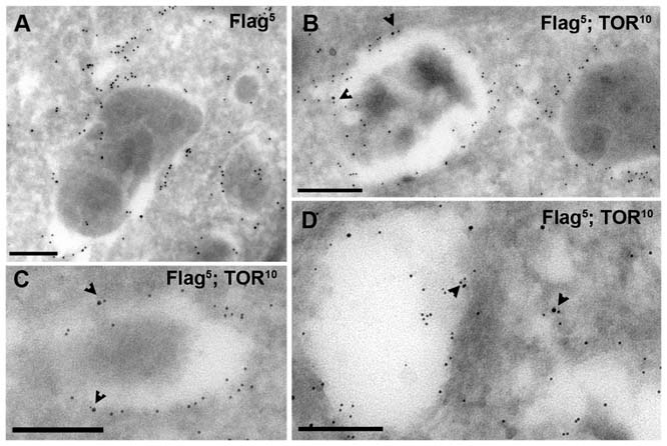
Flag-PAT1 and mTOR co-localise at the surface of the same intracellular compartments. (**A**) Ultrathin sections from a stable HEK-293 cell line overexpressing Flag-PAT1 and either labelled with anti-Flag antibodies (5 nm gold) or (**B–D**) co-labelled with anti-Flag (5 nm gold) and anti-mTOR (10 nm gold) antibodies. Endogenous mTOR and Flag-PAT1 are found on the surface of the same membrane-bound compartments that often contain an electron-dense core. Arrowheads mark a subset of locations where immunolabelled mTOR is in close enough proximity to Flag-PAT1 to be part of a multimolecular complex. Scale bar is 200 nm in all panels.

To determine whether PAT1-containing LEL compartments recruit mTOR upon AA- stimulation, we co-immunostained the Flag-PAT1 cell line with anti-mTOR and anti-LAMP2 antibodies; although Flag-PAT1 and LAMP2 co-localise extensively in the absence or presence of AAs (Figures 1D and 1E), mTOR shuttles to a subset of the LAMP2/PAT1-positive compartments only in the presence of AAs (Figure 1E). Consistent with previous studies [29], [30], mTOR is diffusely expressed throughout the cytoplasm in starved cells and does not localise to LAMP2/PAT1-containing compartments (Figure 1D). Immunogold-labelling and electron microscopy of AA-replete cells, under steady state conditions, revealed the presence of specific membrane-bound compartments. These typically had electron-dense cores, containing both mTOR and Flag-PAT1 (Figure 2). The immuno-positive mTOR molecules were frequently sufficiently close (within 20 nm) to immuno-positive PAT1 molecules to be part of a macromolecular complex. This is consistent with the idea that PAT1 might be involved in the recruitment of mTOR to these membranes or in the regulation of mTOR at the membrane.

### PAT1 and RagC form part of a putative amino acid-sensing complex

**Figure 3:**
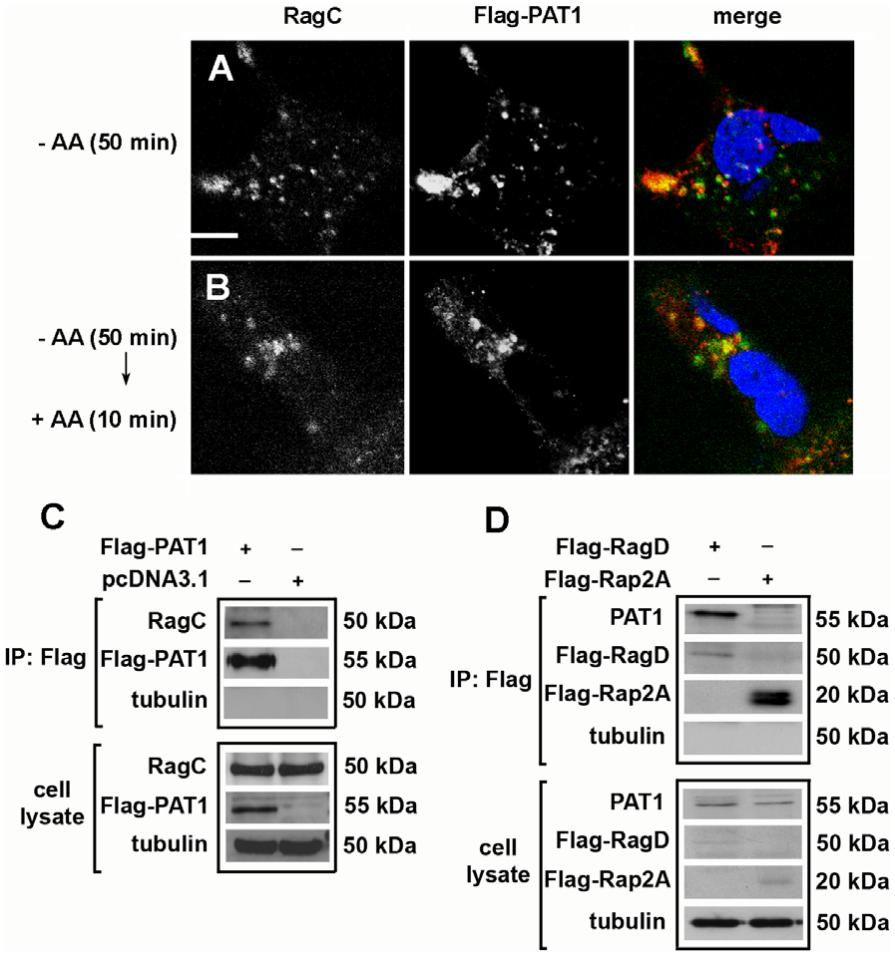
PAT1 co-localises and can physically interact with Rag GTPases. (**A**, **B**) Flag-PAT1 co-localises with a subset of the compartments containing endogenous RagC under both AA-starved (A) and AA-stimulated (B) conditions in a stable HEK-293 cell line overexpressing Flag-PAT1. (**C**) Under steady state conditions, immunoprecipitation of Flag-PAT1 leads to co-immunoprecipitation of endogenous RagC, but not tubulin. (**D**) Conversely, immunoprecipitation of Flag-RagD, but not Flag-Rap2A, both transiently expressed in HEK-293 cells, leads to co-immunoprecipitation of endogenous PAT1, but not tubulin, suggesting that the Rag GTPases complex with PAT1 in cells. Scale bar in A is 10 µm and applies to all panels in A and B.

In yeast, Gtr2p (the orthologue of both RagC and RagD), regulates vesicular shuttling of an amino acid transporter (Gap1) through a direct interaction with this molecule [47]. We found that RagC and Flag-PAT1 co-localise in both AA-starved (Figure 3A) and AA-stimulated conditions (Figure 3B). Under AA-stimulated conditions we observed less overlap between tagged PAT1 and RagC than between PAT1 and LAMP2, suggesting that RagC and PAT1 are co-located in only some LEL compartments. To investigate whether RagC and PAT1 might physically interact, Flag-PAT1 was immunoprecipitated with an anti-Flag antibody from extracts of cells stably expressing the Flag-PAT1 construct. Endogenous RagC was co-immunoprecipitated with Flag-PAT1 (Figure 3C), but was not precipitated from control cell lysates that contained the empty pcDNA3.1 vector. Furthermore, Flag-immunoprecipitated extracts from cells transiently expressing Flag-RagD contained endogenous PAT1, but immunoprecipitated extracts from cells transiently expressing another monomeric G protein, Flag-Rap2A, did not (Figure 3D). We therefore conclude that Rag GTPases not only co-localise with PAT1 in specific LELs, but also form part of a multiprotein complex in these compartments. Since PATs directly interact with AAs, and Rag GTPases are also involved in AA-dependent processes that localise and activate mTORC1, we reasoned that this complex might participate in AA sensing.

**Figure 4:**
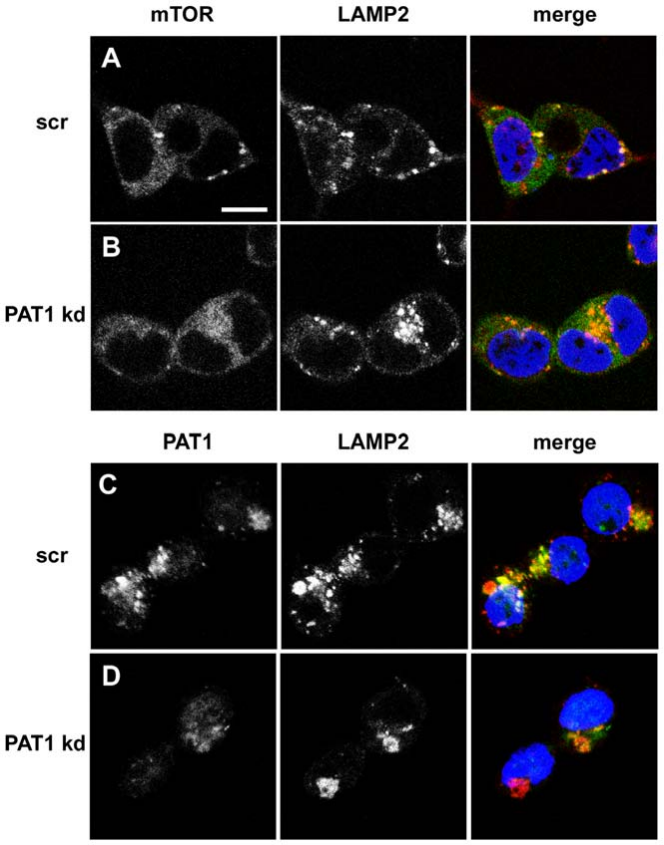
PAT1 modulates the AA-dependent relocalisation of mTOR to LELs. (**A**, **B**) Knockdown of PAT1 (PAT1 kd) in HEK-293 cells reduces the AA-stimulated accumulation of mTOR (green) to LAMP2-positive (red) LELs (B), when compared to cells treated with a scrambled siRNA (scr; A). (**C**, **D**) Importantly, *PAT1* knockdown does not eliminate all PAT1 protein from cells (compare D with control cells in C), so residual mTOR relocalisation in B may result from the presence of low levels of PAT1. PAT1 antibody staining is shown in green, LAMP2 in red. Scale bar in A is 10 µm and applies to all panels.

To test this hypothesis, we investigated whether siRNA knock down of *PAT1*, which we have previously shown severely inhibits mTORC1 activation [46], affects the AA-dependent relocalisation of mTOR in HEK-293 cells. The relocalisation of mTOR was partially but not completely suppressed, under these conditions (Figure 4), indicating that PAT1 is involved in this process.

### GFP-tagged PATs behave similarly to untagged PATs in *Drosophila*

Our analysis in immortalised cells in culture suggests that PAT1 is almost exclusively located on intracellular LELs from where it promotes mTORC1-mediated growth. However, earlier studies in normal mammalian cells *in vivo* suggest that, PATs are distributed between the plasma membrane and intracellular compartments [43], [48] raising the possibility that differential distribution between the cell surface and LELs is involved in regulating PAT activity. We investigated this question in *Drosophila*, where the candidate molecules that control PAT localisation can be more easily genetically modulated *in vivo*. We have previously shown that human PATs are able to promote growth and TORC1 activation in flies [46], suggesting that PAT-dependent regulatory mechanisms are likely to be highly conserved.

To assess the subcellular localisation of PAT transporters in flies, we generated transgenic flies (Methods S1), expressing tagged constructs in which Enhanced Green Fluorescent Protein (GFP) is fused to the C-terminus of the coding sequences of two different PATs (Methods S1) that have previously been shown to have growth-promoting properties, PATH and CG1139 [49]. These constructs, CG1139-GFP and Path-GFP, were expressed in *Drosophila*, in a tissue- and stage-specific fashion, using the GAL4/UAS targeted misexpression system [50]. Analysis of the growth-promoting activity and subcellular localisation of both tagged PATs produced similar results. We primarily present the data for CG1139-GFP below.

We first tested for functional activity of tagged CG1139. Previous studies have shown that low level, ubiquitous expression of CG1139 can rescue the infertility and partially rescue the reduced growth of a recessive *path^KG06640^* mutant [49]. Expression of CG1139-GFP using the arm-GAL4 transgene employed in this previous study, which drives low level ubiquitous expression, resulted in a significant increase in the weight of recessive *path^KG06640^* mutant flies from 0.85±0.02 mg/female fly to 1.02±0.05 mg/fly (P<0.01; normal control females weigh 1.10±0.09 mg/fly), but like untagged CG1139 [49], had no significant effect on wild type flies. Furthermore, mutant females, which are normally infertile, produced offspring in the presence of the tagged PAT. We therefore conclude that CG1139-GFP retains normal *in vivo* functional activity.

**Figure 5:**
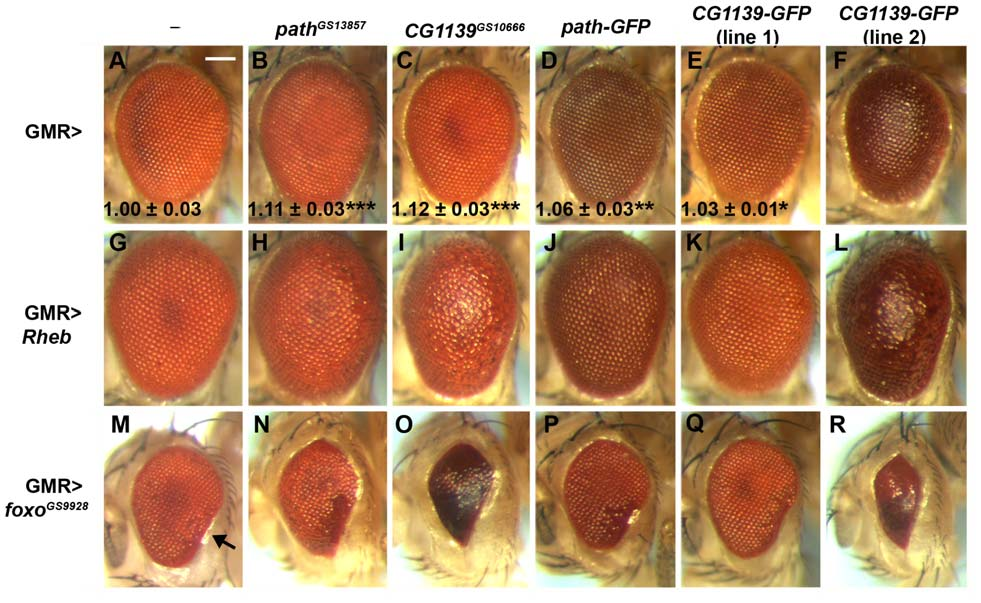
*Drosophila* PAT-GFP fusion proteins have similar functional activities to untagged PATs *in vivo*. Untagged transporters synthesised from GS insertions in *path* (*path^GS13857^*; B, H, N) and *CG1139* (*CG1139^GS10666^*; C, I, O), and tagged transporters synthesised from *UAS-Path-GFP* (D, J, P) and *UAS-CG1139-GFP* line 1 (E, K, Q) and line 2 (F, L, R) insertions were expressed in the differentiating cells of the fly eye with GMR-GAL4 in the presence or absence of other transgenes. (**A**–**F**) Overexpression of *path^GS13857^* (B) and *CG1139^GS10666^* (C) produces a significant increase in ommatidial size relative to controls (A). A more subtle, but also significant, increase in growth was seen for PATH-GFP (D) and CG1139-GFP line 1 (E). CG1139 line 2 produced a bulging and disorganised eye phenotype (F). For size comparisons, n = 6; bottom of panels A–E, mean ± s.d. relative to control; * P<0.05, ** P<0.01, ***P<0.001. (**G**–**L**) The overgrowth induced by overexpressing *UAS-Rheb* with GMR-GAL4 (G) is synergistically enhanced by co-expression of tagged and untagged transporters (H–L), suggesting a role for Rheb signalling in controlling the growth-promoting activity of PATs. (**M**–**R**) The apoptotic, reduced eye phenotype produced by overexpression of *foxo^GS9928^* with GMR-GAL4, which is most clearly seen at the ventro-posterior edge of the eye (arrow in M), is enhanced by co-expression of tagged and untagged transporters (N–R), consistent with these molecules acting through the TORC1 signalling cascade to inhibit Akt and enhance, FOXO activity. Scale bar is 100 µm and applies to all panels. Ommatidial size measurements were made when expression of transgenes did not disturb the ommatidial array.

Using multiple insertion lines, overexpression of the *UAS-CG1139-GFP* and *UAS-path-GFP* constructs in the differentiating eye with GMR-GAL4 generally produced a significant, but more modest, increase in ommatidial size (Figures 5D, E) than UAS-containing constructs driving expression of untagged versions of the PATs (Figures 5B and 5C compared to 5A; [49]). However, one *UAS-CG1139-GFP* line (line 2) gave a bulged eye phenotype, which is commonly observed when growth is strongly stimulated in the differentiating eye (Figure 5F); c.f. [49], [51].

We have previously shown that co-expression in the eye of growth regulatory genes with the transcription factor FOXO, provides a highly sensitive test for TORC1 signalling components [49]. Increased TORC1 activity, which normally promotes growth, suppresses PI3K/Akt signalling via a negative feedback mechanism [52], [53], and this appears to enhance the pro-apoptotic effects of FOXO ([46], [49]; Figures 5N and 5O compared to 5M). All *UAS-PAT-GFP* lines strongly exacerbated the FOXO-induced reduced eye phenotype (Figures 5P–R), even though they generally produced a modest overgrowth phenotype when expressed alone, indicating that the fusion proteins they produce interact similarly to untagged PATs with the TORC1 signalling cascade.

To test whether the UAS-PAT-GFP insertion lines give different phenotypes, because they are expressed at different levels as a result of the chromosomal position of each transgene insertion, we expressed all of these lines in the late third instar larval fat body using the Lsp2-GAL4 driver and measured transcript levels by Q-RT-PCR (Methods S1 and Figure S1). Only the CG1139-GFP line 2, which produces strong phenotypes, was expressed at levels comparable to the UAS-PAT lines we have used in previous studies (Figure 5 and Figure S1 [46]). Although confocal fluorescence microscopy reveals detectable levels of CG1139-GFP and PATH-GFP fusion proteins in the fat body for the other PAT-GFP lines (see below), transcripts from these GFP-tagged constructs are expressed at similar levels to endogenous PATs. We conclude that PAT-GFP fusion proteins are functional *in vivo* and that the weakest expressing lines, particularly CG1139-GFP line 1, which is primarily employed in the analysis presented below, provide powerful tools to assess PAT localisation without producing a strong effect on TORC1 signalling.

### *Drosophila* PATs, like mammalian PATs, are localised to the cell surface and LEL membranes in multiple cell types

**Figure 6:**
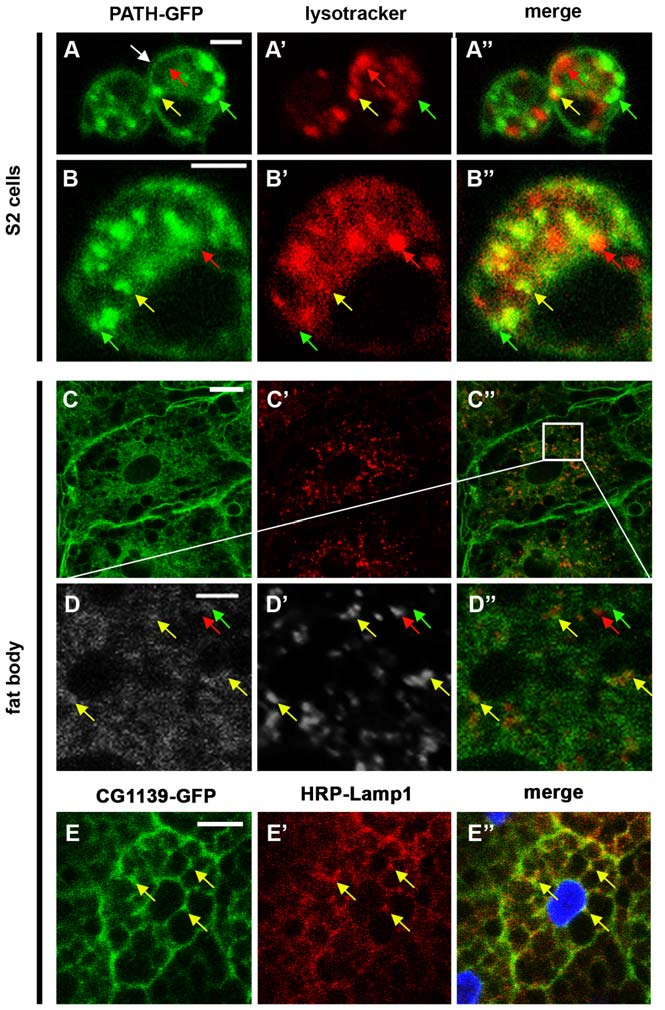
*Drosophila* PATs are localised at the cell surface and on LEL compartments *in vivo*. (**A**, **B**) Expression of PATH-GFP (green) in living *Drosophila* S2 cells stained with the acid-sensitive dye Lysotracker Red. Some PATH-GFP is found at the plasma membrane (white arrow), but most is at the surface of intracellular compartments. It does not generally co-localise with the most intensely stained Lysotracker Red-positive compartments (likely to be lysosomes, e.g. red arrows), but does co-localise with the less intensely stained Lysotracker Red-positive compartments (probably late endosomes and some lysosomes, e.g. yellow arrows), perhaps because GFP fluorescence or integrity is affected in highly acidic conditions. In addition, some PATH-GFP-containing compartments do not co-stain with Lysotracker Red (e.g. green arrows). (**C**, **D**) PATH-GFP (green) expressed under Lsp2-GAL4 control in the larval fat body also co-localises with only a subset of Lysotracker Red-positive compartments in living tissue (e.g. examples marked with arrows as in A and B), but is also expressed at high levels at the surface of cells. Note in D, PATH-GFP is specifically expressed at the surface of some larger Lysotracker Red-positive structures (yellow arrows) and other membrane structures (green arrows), consistent with its known membrane-association. (**E**) CG1139-GFP co-localises with many, but not all compartments stained with HRP-Lamp1, a late endosomal and lysosomal marker, in fixed larval fat bodies (e.g. yellow arrows), suggesting that some, but not all, intracellular CG1139 is in LELs. Nuclei are stained with DAPI (blue) in E″. Scale bar is 5 µm in A, B and D, and 20 µm in C and E.

The PAT-GFP open reading frames (ORFs) were cloned into a metallothionein-inducible vector to permit expression in *Drosophila* Schneider 2 (S2) cells. Even in the absence of copper induction, the fusion proteins were produced at detectable levels. However, relatively few transfected cells with normal morphology were observed with the CG1139-GFP construct, suggesting a toxic effect when overexpressed in this system. We therefore focused our analysis on PATH-GFP in this cell type. This fusion protein was located mainly on intracellular organelles (e.g., Figures 6A and B), with limited cell surface expression. Many, but not all, of the GFP-positive intracellular organelles were also labelled with LysoTracker Red, which stains acidic lysosomes and at least some late endosomes, in living cells. However, the majority of the largest organelles that stained most strongly with Lysotracker Red, which are likely to be lysosomes, were not GFP-positive. Based on the proposed topology of PAT1 [54], the C-terminal GFP tag on the PATH and CG1139 fusion proteins employed in this study would be predicted to lie on the intralumenal face of the intracellular compartments. It is therefore very likely that the GFP tag is degraded or inactivated in lysosomes, explaining the absence of GFP in these organelles in *Drosophila* when compared to endogenous PAT proteins in mammalian cells.

To assess the localisation of PAT-GFP in living tissue, the fusion constructs were expressed using the Lsp2-GAL4 driver [55] in the larval fat body, which contains large cells that have previously been used to study the subcellular localisation of AATs [22]. The intracellular distribution of PATs in living cells with respect to Lysotracker Red staining was comparable to S2 cells (e.g., PATH-GFP in Figures 6C and D), although there was an increased level of general cytoplasmic GFP staining observed and substantial plasma membrane expression. GFP was localised at the surface rather than in the lumen of the largest organelles (Figure 6D), consistent with its membrane localisation.

To determine more precisely in which intracellular organelles the fusion proteins reside, we co-stained larval fat bodies with antibodies against proteins that mark specific subcellular compartments (Methods S1). Although there was only limited co-localisation with markers for early endosomes, which are typically localised to a perinuclear region in the fat body (e.g. see Figure S2 [56]), most of the GFP-positive organelles also expressed an HRP-Lamp1 fusion protein (yellow arrows in Figure 6E) that is primarily found on the surface of LELs [56]. We conclude that under normal physiological conditions in living flies, the PATs reside at the cell surface and on the membranes of LELs within fat body cells, whereas in Schneider cells in culture, PATs are largely at the surface of LELs.

### PI3K/Akt/Rheb signalling stimulates the endocytosis of PATs and enhances their growth-promoting properties

Our previous analysis of cells mutant for a hypomorphic *path* allele suggested that PATH plays a more important role in cell-autonomous growth regulation in cells where signalling by Rheb, a monomeric G protein that can act downstream of PI3K/Akt to positively regulate TORC1 activity [57], [58], is elevated [49]. To further investigate this link, tagged and untagged PATs were co-expressed using the GMR-GAL4 driver in the differentiating eye with Rheb. The growth-promoting effects of the PATs, particularly those that when expressed alone produced obvious overgrowth phenotypes, were strongly enhanced in the presence of Rheb (Figures 5H–L compared to 5B–F and 5G). However, even the weakest PAT-GFP lines produced a more bulged and disorganised eye.

**Figure 7:**
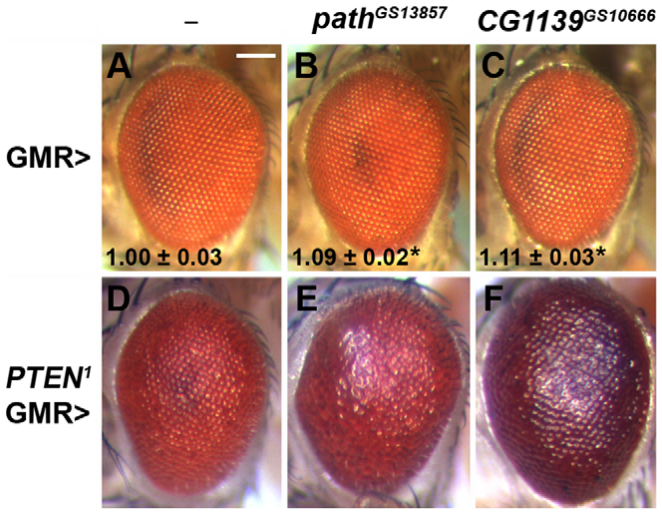
The growth-promoting activity of PAT transporters is synergistically enhanced by hyperactivation of PI3K/Akt signalling. (**A**–**C**) GAL4-UAS-induced overexpression of the fly PAT transporter genes, *path* (B) and *CG1139* (C) in the differentiating eye with GMR-GAL4 promotes increased growth compared to normal animals (A). (**D**–**F**) When overexpressed in a *PTEN* mutant background (D), the effect of *path* (E) and *CG1139* (F) on growth is synergistically enhanced, resulting in a highly overgrown, bulging eye phenotype. Ommatidial size measurements are given for eyes where the ommatidial array is regularly arranged (n = 6; bottom of panels A–C, mean ± s.d. relative to control (A); *P<0.001, increased relative to control). Fly genotypes are *w*; *GMR-GAL4* (A), *w*; *GMR-GAL4*/*path^GS13857^* (B), *w*; *GMR-GAL4*/*CG1139^GS10666^* (C), *y w*; *PTEN^1^ FRT40A*/*P[w^+^]l(2)3.1 FRT 40A*; *GMR-GAL4* (D), *y w*; *PTEN^1^ FRT40A*/*P[w^+^]l(2)3.1 FRT 40A*; *GMR-GAL4*/*path^GS13857^* (E) and *y w*; *PTEN^1^ FRT40A*/*P[w^+^]l(2)3.1 FRT 40A*; *GMR-GAL4*/*CG1139^GS10666^* (F).

Akt can indirectly activate Rheb by blocking the inhibitory effects of the Tuberous sclerosis Complex (TSC; [59]). To test whether upregulating PI3K/Akt signalling in the eye could also modulate the growth-promoting activity of the PATs (Figures 7B and C compared to 7A), we generated eyes that were almost entirely mutant for the key antagonist of this pathway, the fly homologue of the major tumour suppressor gene, *PTEN* (Figure 7D; [17], [60]), using the *ey*-FLP/FRT somatic recombination system and a recessive cell lethal chromosome [61]. There was a strong synergistic interaction between *PTEN* and both the *CG1139* and *path* transporter genes. *PTEN* mutant eyes expressing these molecules under GMR-GAL4 control were more bulged compared to non-expressing eyes, and contained visibly larger and more disorganised ommatidia (Figures 7E and 7F compared to 7B–D). Thus, PI3K/Akt/Rheb signalling synergises with overexpressed PATs to promote *in vivo* growth in the eye.

**Figure 8:**
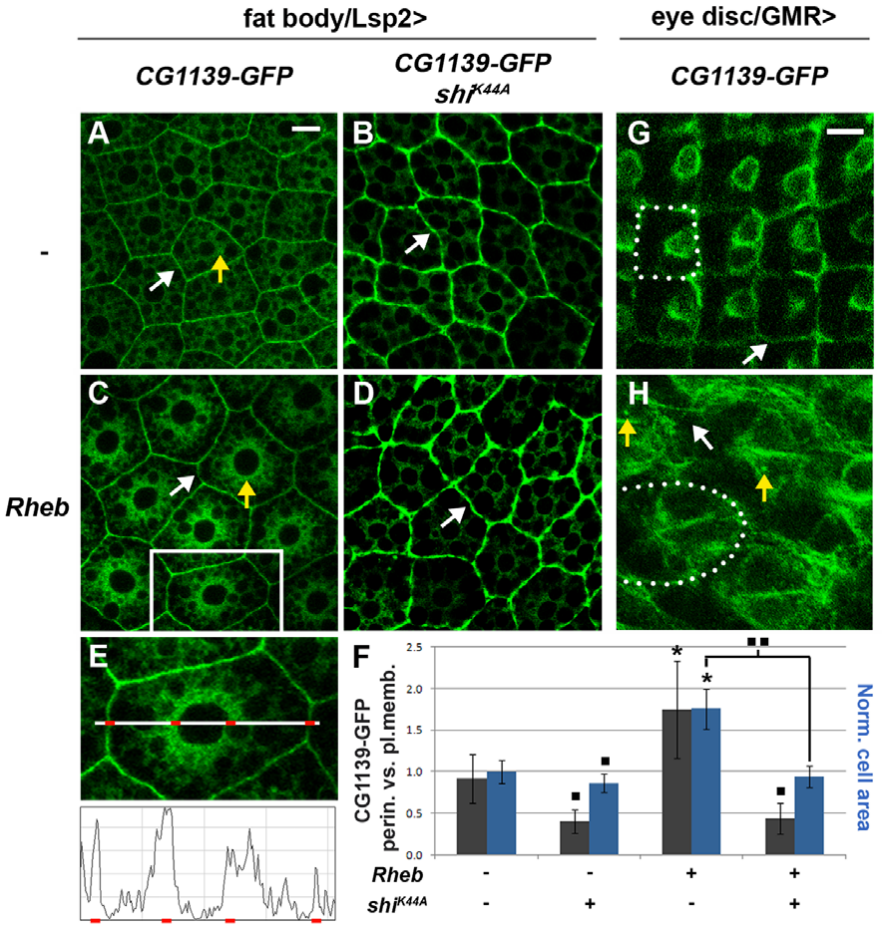
PI3K/Akt/Rheb signalling promotes shuttling of the PATs to endosomal compartments. (**A, C**) CG1139-GFP expressed in the larval fat body using Lsp2-GAL4 is localised to both the plasma membrane (white arrow) and also to intracellular LELs throughout the cytoplasm (yellow arrow; A). Overexpression of Rheb leads to an increase in the relative proportion of intracellular, including perinuclear, protein compared to cell surface CG1139-GFP (C). (**B**, **D**) When CG1139-GFP is co-expressed with a dominant negative version of Shibire, Shi^K44A^, which blocks endocytosis, in either the presence (D) or absence (B) of Rheb, the transporter is mostly located at the plasma membrane (white arrow), strongly suggesting that PATs are normally shuttled to LELs from the cell surface. (**E**, **F**) The ratio of the GFP signal intensity in a 2.25 µm perinuclear region and a 2.25 µm region at the plasma membrane (see E) was measured for genotypes in A–D (grey bars; error bars = s.d. in F). Average cell size for each genotype is also shown in F (blue bars; error bars = s.d.). n = 25; *P<0.001 (increased) and _■_ P<0.001 (decreased) relative to non-Rheb/non-Shi^K44A^-expressing control; _■ ■_ P<0.001, decreased relative to Rheb-overexpressing control. Rheb-induced changes in intracellular PATs and PAT-induced growth are entirely dependent on endocytosis. (**G**, **H**) In the larval eye imaginal disc, CG1139-GFP, expressed under GMR-GAL4 control (G), is mostly located at or near the plasma membrane, for example at the surfaces of flattened non-photoreceptor cells that surround each ommatidium (white arrows). Co-overexpression of Rheb (H) results in much larger ommatidia, with reduced staining around the ommatidial border (white arrow) and more CG1139-GFP cytoplasmic expression, including intense intracellular punctae of staining (yellow arrows), consistent with Rheb promoting endocytosis of PATs in this tissue. An outline of an individual ommatidium in G and H is marked with a dashed line. Scale bar in A is 20 µm and applies to panels A–D, scale bar in G is 5 µm and applies to panels G and H.

Hennig *et al*., 2006 previously showed that elevated Rheb signalling in the fat body promotes bulk endocytosis, and provided evidence that bulk and selective endocytic regulatory events act both upstream and downstream of TORC1 signalling. We tested whether the synergistic interaction between PI3K/Akt/Rheb and the PATs might be linked to a change in the subcellular localisation of the PATs. Co-expression of the PATs with Rheb using Lsp2-GAL4 significantly increased the size of fat body cells by 1.76±0.24 fold relative to cells only expressing the PAT (1.00±0.14; P<0.001; Figures 8C and 8F), and altered the subcellular distribution of the PATs, which accumulated at much higher levels within cells, including a region around the nucleus (compare Figure 8A to 8C). Fluorescence intensity measurements specifically in this perinuclear region compared to the plasma membrane revealed a highly significant approximately two-fold increase in perinuclear GFP expression when Rheb is overexpressed with CG1139-GFP (Figures 8E and 8F). Since the HRP-Lamp1 fusion protein is localised in structures positioned throughout the cytoplasm both in the absence and presence of Rheb (Figures S2C and S2D), this specific pool of perinuclear CG1139 is probably not located in LELs. However, early endosomes, marked by a *UAS-GFP-FYVE* transgene [62], are found in a perinuclear region in this tissue [56] and GFP-FYVE-positive structures are also perinuclear in Rheb-overexpressing fat bodies (Figure S2B), suggesting that some of the PATs are most likely endocytosed to early endosomes when Rheb is overexpressed, as well as to other structures that are not perinuclear, which are likely to be LELs.

To test whether Rheb-dependent endocytosis of CG1139 is critical for the synergistic interaction between these molecules, CG1139-GFP was co-expressed in the fat body both in the absence and presence of Rheb with an inhibitory form of the dynamin homologue Shibire, Shi^K44A^ [63], which blocks endocytosis (Figures 8B and 8D respectively). Intracellular levels of CG1139-GFP were significantly reduced in both cases to similar levels (Figure 8F), showing that the accumulation of intracellular PATs in fat body cells in the presence or absence of Rheb overexpression requires endocytosis from the cell surface. Interestingly, blocking Shibire function also completely and selectively suppressed the cell size increase induced by Rheb and PAT co-expression (Figure 8F), demonstrating that endocytosis is an important contributor to the synergistic growth-promoting functions of these two molecules in fat body cells.

To investigate whether Rheb's effects on PAT subcellular localisation are conserved in other cell types, we expressed CG1139-GFP in the differentiating eye using the GMR-GAL4 driver in the absence or presence of Rheb. Co-expression of Rheb and CG1139-GFP produced a large increase in adult ommatidial size (Figure 5K compared to 5G and 5E) and in photoreceptor size in the larval eye imaginal disc (compare Figures 8H to 8G). In the absence of Rheb, CG1139 was highly expressed at the cell surface, as evidenced by the ring of staining associated with the membranes of non-photoreceptor cells around each ommatidial unit (Figure 8G). This staining was less pronounced in eyes overexpressing Rheb, with each photoreceptor cell having more diffuse non-nuclear intracellular GFP (Figure 8H). In addition, intense punctae of intracellular staining were observed in these latter cells (Figure 8H), consistent with our interpretation that PATs are endocytosed in response to increased Rheb signalling. Overall we conclude that PI3K/Akt/Rheb signalling enhances the growth-promoting properties of the PATs and that this process requires endocytosis. Since we have shown in mammalian cells that LEL-located PATs are involved in mTORC1 activation, the synergistic growth regulatory interactions between PI3K/Akt/Rheb signalling and PATs are presumably at least partly explained by increased accumulation of PATs in LELs.

## Discussion

The two ubiquitously expressed proton-assisted AATs, PAT1 and PAT4, have previously each been shown to act as essential mediators of AA-dependent mTORC1 activation in rapidly growing HEK-293 cells, even though at least one of these molecules, PAT1, is almost exclusively intracellular [46]. In this paper, we demonstrate that in these cells intracellular PAT1 is primarily located in LELs and that it forms a complex with the Rag GTPases, molecules that are also involved in AA-dependent mTORC1 activation. Our data suggest that this PAT1/Rag/Ragulator complex plays a key role in the process of AA-sensing that regulates Rag-dependent mTOR relocalisation to the LELs, and activates mTORC1 signalling. Although PAT4 is also required for AA-stimulated TORC1 activation [46], we have not yet been able to determine whether it is also part of this complex. Importantly, PATs are found at both the cell surface and LELs in living flies. Altering PI3K/Akt1/Rheb signalling *in vivo* promotes endocytosis of PATs, a process required to produce synergistic growth regulatory interactions between these molecules. These findings partly explain how signals from growth factors, extracellular nutrients and LELs might interact together to maximise mTORC1 activation [26].

### PAT1 complexes with Rag GTPases and mediates amino acid-dependent mTORC1 activation from LEL compartments

The data presented in this study highlight a critical role for PATs on the surface of LELs in regulation of TORC1/S6K signalling. Several previous studies have shown that PATs can be located at the cell surface and/or the surface of LELs and that their distribution is cell type-specific [64]–[67]. In rapidly growing cells, such as HeLa cells [48], MCF7 and HEK-293 cells [46], PAT1 is almost exclusively localised on intracellular membrane-bound structures, which we now demonstrate for HEK-293 cells are LAMP2-positive LELs. In the fly fat body, GFP-tagged versions of CG1139 and PATH, two fly growth-regulatory PATs, partially co-localise with LEL markers. In contrast to rapidly proliferating *Drosophila* S2 cells in culture, a significant proportion of these transporter proteins are also located at the plasma membrane, where these proteins presumably cannot modulate TORC1 activity via the LEL-dependent mechanism observed in mammalian cell culture.

**Figure 9:**
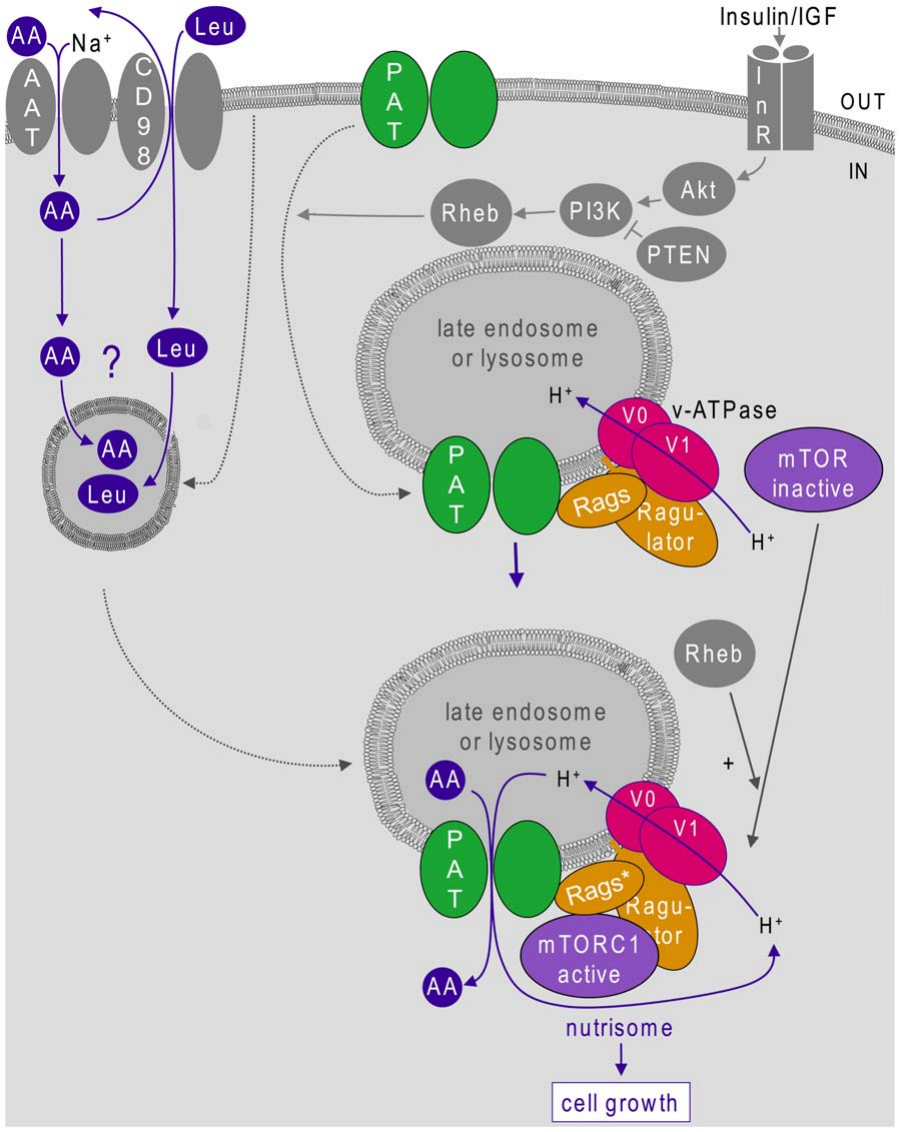
AA-dependent regulation of mTORC1in late endosomes and lysosomes via the PAT1/Rag/Ragulator/v-ATPase nutrisome complex. We have shown that Proton-assisted Amino acid Transporters (PATs) localised on late endosomes and lysosomes (LELs) interact with Rag GTPases (Rags) and are required for mTORC1 activation. The Ragulator is a trimeric group of proteins involved in attaching the complex to the lipid bilayer. Recent studies [41] have also demonstrated that the vacuolar H^+^-ATPase (v-ATPase) interacts with the activated Rag (Rag*)/Ragulator complex to control amino acid (AA)-dependent mTORC1 activation and that this is regulated by the rapid accumulation of extracellular AAs in LELs. This suggests a model where, in response to AAs (compare upper LEL to lower AA-stimulated LEL), these different molecules form a complex that we call the ‘nutrisome’. Cycling of protons through this nutrisomal engine induces conformational changes that activate mTORC1, leading to increased translation and cell growth. Importantly, signalling from the insulin receptor (InR) and subsequent activation of the PI3K/Akt/Rheb cascade promotes shuttling of PATs from the cell surface to LEL membranes, hence increasing PAT-dependent mTORC1 activation and cell growth. In addition, the accumulation of AAs in the LEL lumen presumably involves transport into intracellular endosomal compartments (depicted by compartment on left hand side) via currently unknown amino acid transporters (AATs) or potentially endocytosis. Cytoplasmic leucine (Leu), which may be brought into cells via the heterodimeric AAT, CD98 [34], has been shown to play a key role in activating mTORC1 in some cultured cells and may play a key role in this process. Influx of Leu or other AAs into the endolysosomal system may ultimately allow the AA substrates of PAT1 to accumulate in the LELs through AA exchange mechanisms, leading to PAT1-mediated activation of the nutrisome. Interactions leading to activation or inhibition of downstream components are depicted by solid arrows and solid bars respectively, movement of specific molecules by dashed arrows and processes involving membrane shuttling by dotted arrows.

PATs are, to date, the only intracellular molecules implicated in AA-dependent mTOR activation that are known to bind to AAs. Our findings that PAT1 is located in LELs, some of which recruit mTOR upon AA stimulation, and that PAT1 co-immunoprecipitates with Rag GTPases, other key molecules that play an evolutionarily conserved role in this process [28]–[30] strongly suggest that the PAT1/Rag/Ragulator complex functions in some form of AA-sensing mechanism operating in specific LELs that is critical for mTORC1 activation (Figure 9).

Indeed, the close proximity of membrane-associated immunoreactive mTOR and PAT1 molecules in our EM studies and the effect of *PAT1* knock down on AA-dependent mTOR relocalisation are consistent with a model in which this PAT1/Rag complex can recruit mTOR directly. Although the PAT1 knock down experiments did not completely block mTOR relocalisation (compare Figures 4A and 4B), we found that this approach did not completely remove all *PAT1* mRNA and protein (compare Figures 4C and 4D; [46]), and so it is possible that low levels of PAT1 can still recruit some mTOR to LELs, but cannot activate it normally. Alternatively, there may be other AA-binding molecules that can partially substitute for PAT1 in this process or that play an important role in initially recruiting mTOR to the LELs.

### Regulation of PAT trafficking and its role in growth regulation

To study the subcellular localisation of the PATs *in vivo*, we employed GFP-tagged forms of PATH and CG1139 that are functionally active, but expressed at low levels, to minimise their effect on the TORC1 signalling cascade, which is known to stimulate endocytosis in flies [22]. Indeed, most of the CG1139-GFP fusion protein produced by the strongly expressing line 2 (Figure 5) is predominantly found in endosomes and lysosomes, even under normal Rheb signalling conditions (data not shown), consistent with the idea that when highly expressed, these molecules can self-regulate their subcellular localisation via TORC1. Co-expression of these tagged PATs in the fat body with dominant negative Shibire, which blocks endocytosis, leads to accumulation of these molecules at the cell surface, eliminating the possibility that these fusion proteins are abnormally trapped during synthesis and processing in the ER or Golgi, or inappropriately by pass the plasma membrane on their way to LELs.

In this *in vivo* system, elevated PI3K/Akt/Rheb signalling not only promotes PAT-mediated growth synergistically, but increases the proportion of perinuclear to plasma membrane PATs, consistent with the idea that intracellular PATs are critical for growth control. None of our data exclude the existence of TORC1-regulatory mechanisms involving other subcellular compartments. Nor do they exclude the possibility that other mechanisms could link PI3K/Akt with mTORC1 signalling at LELs (e.g., Rheb interaction with TORC1 [30] or the regulation of pH in LELs [68]). But collectively our data do indicate that PATs and growth-regulatory TORC1 signalling from LELs become critically important as PI3K/Akt/Rheb signalling is increased. Indeed, although blocking endocytosis only slightly inhibits normal growth of the fat body, it completely suppresses Rheb-induced overgrowth (Figure 8). Hennig and co-workers have previously presented evidence that endocytosis modulates TORC1 signalling in flies, having both positive and negative effects in different developmental scenarios [22]. Our data are consistent with their idea that this function may be linked to shuttling of different nutrient transporters. Indeed, our studies suggest that the critical AATs involved in TORC1 activation may change as PI3K/Akt/Rheb signalling is elevated, with PATs playing an increasingly important role, hence explaining how endocytosis can modulate TORC1 in a context-dependent fashion.

As already discussed above, PATs may function in the mTORC1 activation process by sensing intralumenal AAs in LELs. This might be particularly important when the PI3K/Akt/Rheb pathway is hyperactivated, as in cancer, since cells could shield themselves from changes in extracellular AA levels by increasing their dependence on AA-dependent PAT signalling in LELs, providing a partial explanation for the known growth advantage of these cells, even in starvation conditions [24].

It has recently been demonstrated that during autophagy, cultured human cells employ AAs in the lumen of the autolysosome to activate mTORC1 and promote survival [42]. The process of autophagy is also critical for the survival of cancer cells exposed to stresses such as hypoxia [69], [70]. It will now be interesting to investigate whether PATs are involved in these processes. If they are, PATs may represent novel and selective drug targets for inhibiting growth of cancer cells under these conditions.

### A new model for mTORC1 regulation in LELs via the PAT-containing nutrisome complex

Since PATs are proton-dependent amino acid transporters that can potentially export AAs out of LELs, the simplest explanation for their mTORC1 regulatory activity is that PAT-dependent, proton-mediated AA transport out of the LELs is required to activate mTORC1 signalling at the LEL membrane. A recent report has identified the vacuolar H^+^-ATPase (v-ATPase) as an additional component of the Rag/Ragulator complex that senses intralumenal AAs. These AAs rapidly accumulate inside LELs in response to addition of extracellular AAs, and then recruit and activate mTORC1 on LELs [41]. Perhaps, the most straightforward explanation of these and our data is that PATs and the v-ATPase function as an AA-sensing engine (or ‘nutrisome’) at the LEL membrane (Figure 9). PATs transport AAs and protons out of the LELs, and the coupled v-ATPase pumps the protons back into the LEL lumen. Zoncu et al. (2011) have already established that the v-ATPase undergoes altered interactions with the Rag/Ragulator complex in response to AAs. It, therefore, seems likely that AA-driven nutrisome activity induces conformational changes in either this molecule, or the PATs or both, that relay a signal to the Rag GTPases to regulate mTORC1 activity.

Although studies *in vivo* and in cell culture [46], [49] have shown unequivocally that PATs drive growth and mTORC1 activation, high level overexpression can inhibit these processes in the same systems [41], [49]. Since much of the overexpressed PAT protein is unlikely to be coupled to Rag GTPases and merely drains the LEL lumen of specific AAs that are substrates for PATs, these data are consistent with a model where the PAT/Rag/Ragulator-v-ATPase complex is required to establish a microenvironment for cycling of protons and export of AAs that is needed to drive the AA-sensing nutrisome. The coupling of v-ATPase and PATs may also explain why reducing the proton gradient, though not the electrochemical potential, across the LEL membrane by treatment with the ionophore FCCP does not block AA-dependent mTOR relocalisation [41].

This model provides a first mechanistic explanation of how mTORC1 senses AAs and a useful framework for more detailed analysis of the AA-sensing mechanisms involved. For example, it will now be important to test the importance of PAT transporter activity in mTORC1 activation, since PATs may also be able to signal to downstream targets via a proton-stimulated transceptor mechanism [16]. Furthermore, the fact that human PAT4 and one of the fly PAT transporters, PATH, which both promote mTORC1-dependent growth, are low capacity, proton-independent, high affinity transporters [49], [71], suggests that these transporters may play additional roles in mTORC1 regulation. It is also of interest that the mechanisms that target AAs to the LELs are completely unknown. Such mechanisms might involve AAs like leucine, which is not a substrate for PAT1, but appears to play an important role in AA-dependent mTORC1 activation, since it could be important for AA exchange processes that target other AAs to LELs. Whatever subsequent studies reveal, the data presented here highlight the LELs and PAT transporters as critical players in AA sensing and potentially important new targets in specifically inhibiting cancer cell growth.

## Materials and Methods

### Culture of human cells

HEK-293 cells (ATCC), were cultured in MEM (Minimum Essential Medium Eagle; Sigma-Aldrich) supplemented with 10% fetal bovine serum (Gibco Invitrogen, Paisley, UK), 1×NEAA (Sigma-Aldrich, Poole, UK) and 4 mM L-Glutamine (Gibco Invitrogen), in the presence of 1% Penicillin/Streptomycin (Invitrogen) at 37°C, 5% CO_2_. Cells were either maintained in this medium (steady state conditions) or AA-starved and in some cases AA-stimulated for 10 min as described in [46], except that 4 mM glutamine was added to the AA stimulation medium.

### Generation of stable HEK-293 cell lines

Stable cell lines carrying either a pcDNA3.1(+) (Invitrogen) construct containing Flag-PAT1 or the empty pcDNA3.1(+) vector were generated as described in [46].

### *Drosophila* S2 cell culture

For immunostaining, *Drosophila* S2 cells were transfected with *pMT/V5-HisB-path-EGFP* using FuGENE HD (Roche, Welwyn Garden City, UK; 3 µl FuGENE to 1 µg DNA) in the presence of 100 µM CuSO_4_ and stained four days later (see below).

### PAT1 knockdown experiments

PAT1 was knocked down in HEK-293 cells using MaTra transfection as described in [46]. Two different siRNAs (si158, si159) were used in different experiments and produced similar results. Cells were analysed by immunostaining 72 h after transfection according to method outlined in [30].

### Immunostaining

The following primary antibodies: mouse anti-Flag; (1∶200; Abcam, ab18230), rabbit anti-mTOR (7C10; 1∶200; Cell Signaling Technology, CST), rabbit anti-RagC (1∶100; CST), rabbit anti-PAT1 (1∶1,000; [48]), rat anti-LAMP2 (1∶100; Abcam ab13524) and mouse anti-LAMP2 (1∶100; Abcam ab25631) and secondary antibodies raised in donkey (1∶500; Jackson ImmunoResearch) were used. Cells were stained according to the procedure described in [30]. Samples were then mounted in Vectashield with DAPI (0.1 µg/ml; Vector Laboratories) and imaged on a Zeiss 510 confocal microscope. Fat body sample preparation for confocal analysis is described in Supplemental data (Methods S1).

### Co-immunoprecipitation

HEK-293 cells were transiently transfected one day after plating in 6-well plates using MATra transfection, as described in [46] with Addgene plasmid 19316 (Flag pLJM1 RagD) or Addgene plasmid 19311 (Flag pLJM1 Rap2A) and incubated for two days prior to lysis. Stable HEK-293 cell lines containing either the Flag-PAT1 construct or the empty pcDNA3.1(+) vector were plated two days before lysis in 6-well plates. Cells were lysed with ice-cold RIPA buffer containing a protease inhibitor cocktail (1∶100; Sigma-Aldrich) for 30 min at 4°C with gentle rocking. Cell lysates cleared by centrifugation (10 min at 42,000 g; (Beckman J2-HS Centrifuge) at 4°C were processed with the FlagIPT-1 kit (Sigma-Aldrich) according to the manufacturer's instructions and then analysed alongside input controls by SDS-PAGE.

### *Drosophila* strains, crosses and phenotypic analysis

The following fly strains were used: *UAS-shi^K44A^* [63], *UAS-HRP-Lamp1* [56], *UAS-GFP-FYVE* [62], *PTEN^1^* [60], *UAS-Rheb* [57], GMR-GAL4, Lsp2-GAL4, arm-GAL4 (Bloomington Stock Center) and the GS lines [72] *path^GS13857^*, *CG1139^GS10666^* and *foxo^GS9928^* (gifts from Toshiro Aigaki). Other fly stocks, genetic crosses and the method for ommatidial size measurement were as described in [49]. Flies were cultured on standard cornmeal agar food at 25°C.

### Western blotting

Crude RIPA lysates from human cells were cleared by centrifugation (13,000 rpm; 10 min; 4°C). In this case, sample concentration was determined using Bradford protein quantification (BioRad) and about 40 µg of boiled protein were routinely loaded onto a 10% SDS polyacrylamide gel and transferred to nitrocellulose membrane (Whatman). Membranes were blocked in TBS+0.1% Tween ([v/v]; TBST)+5% BSA or non-fat dry milk for 1 h, and washed 3×5 min in TBST before overnight incubation in primary antibody. The following primary antibodies were used: anti rabbit anti-RagC (1∶1,000; CST) in 5% BSA, mouse anti Flag M2 (1∶500; Sigma Aldrich) in 3% non-fat dry milk, rabbit anti-Flag (1∶1000; CST) in 5% BSA, rabbit anti-PAT1 (1∶10,000; [48]) in 5% non-fat dry milk and mouse anti-α-tubulin (1∶10,000; Sigma-Aldrich) in 5% non-fat dry milk. Membranes were washed 3×5 min, incubated for 1 h in HRP-conjugated secondary antibodies (1∶4,000; Jackson ImmunoResearch) and washed 3×5 min in TBST before ECL detection (Amersham).

### Lysotracker staining

For lysotracker staining, fat bodies of late third instar larvae were dissected in Schneider's medium (Gibco), incubated for 2 min in 10 µM LysoTracker Red DND-99 (Molecular Probes) in PBS, mounted in Schneider's medium on glass slides and imaged immediately. For lysotracker staining in S2 cells, medium was replaced with fresh medium (pre-warmed) containing 100 nM Lysotracker Red DND-99, mounted directly on glass slides and imaged within 5 min on a Zeiss 510 confocal microscope.

### Analysis of CG1139-GFP subcellular localisation and cell size measurements within fat body cells

Fat bodies from the anterior ends of third instar larvae were dissected in 4% paraformaldehyde in PBS and incubated for 20 min at 4°C before washing for 3×5 min in PBST (PBS+0.3% [v/v] Triton X-100) and staining by standard methods. They were mounted using VectaShield containing DAPI (Vector Laboratories). To determine the subcellular distribution of CG1139-GFP and cell size, five fat bodies were analysed per genotype and five random cells were imaged at the same magnification from each fat body on a Zeiss 510 confocal microscope. For CG1139-GFP localisation measurements, a horizontal line was drawn through the middle of each cell and a pixel value profile plotted using ImageJ. This was done for both CG1139-GFP (488 nm) and DAPI (405 nm) to locate the nucleus on the CG1139-GFP plot. The area under the pixel value curve for CG1139-GFP was calculated for the two 2.25 µm regions just outside the nuclear membranes and for two 2.25 µm regions including the plasma membrane (see Figure 5E). Using these values, the ratio of perinuclear versus plasma membrane signal intensity was calculated. For cell size measurements, each cell was outlined using ImageJ. The average area value was then normalised against CG1139-GFP-expressing control cells. Significance values were calculated using a Student's unpaired two-tailed t-test.

## Supporting Information

Figure S1 **PAT-GFP insertions are expressed at different levels** ***in vivo*** **.** Different *UAS-PAT-GFP* insertion lines and the two Gene Search insertion lines [72] *CG1139^GS10666^* and *path^GS13857^*, were expressed using the Lsp2-GAL4 driver [50], [55], which produces detectable fusion protein in the fat body with all PAT-GFP lines tested (e.g., Figure 6). Levels of *CG1139* (A) and *path* (B) transcripts in fat body RNA preparations were measured using Q-RT-PCR and normalised relative to the levels of the *RpL32* housekeeping control transcript. These data revealed a correlation between levels of PAT expression and the growth-promoting activity of specific constructs. The *CG1139-GFP* line 2 insertion, which gives strong overgrowth and FOXO-dependent cell death phenotypes (Figures 5F, L and R), produced comparable transcript levels to *CG1139^GS10666^*, which we have previously employed to overexpress this transporter at high levels (Figure 5C; [49]). *path* transcripts are normally expressed at moderate levels in the fat body [73]. Levels of *path* transcripts increased when *path-GFP* was expressed using the Lsp2-GAL4 driver, but only to about half the level produced by *path^GS13857^*, which gives stronger phenotypes when overexpressed in the eye (Figures 5B, H and N). * (P<0.001) indicates significantly higher levels than Lsp2>control. _■_ (P<0.001) indicates significantly lower levels of the fusion transcript than with Lsp2>*path^GS1385^*.
(TIF)

Figure S2 **Expression pattern of endosomal markers in the** ***Drosophila*** **larval fat body.** (A–D) Figure shows expression of the FYVE-GFP (early endosomal; A, B) and HRP-Lamp1 (late endosomal; C, D) markers in the larval fat body. In the presence (B, D) and absence (A, C) of Rheb, FYVE-GFP (green) is largely confined to a perinuclear region (arrows), while HRP-Lamp1 (red) has a more widespread punctate distribution throughout the cytoplasm. Nuclei are stained with DAPI (blue). Scale bar in A also applies to B and scale bar in C also applies to D; both are 20 µm.
(TIF)

Methods S1 **Subcloning, production of transgenic fly lines, measurement of mRNA levels in** ***Drosophila*** **fat bodies, fat body immunostaining.**
(DOC)
