# Supplementary figures and images for: Proton-Assisted Amino Acid Transporter PAT1 Complexes with Rag GTPases and Activates TORC1 on Late Endosomal and Lysosomal Membranes
Source: PLoS One. 2012 May 4;7(5):e36616. doi: 10.1371/journal.pone.0036616 (PMC3344915; doi:10.1371/journal.pone.0036616)

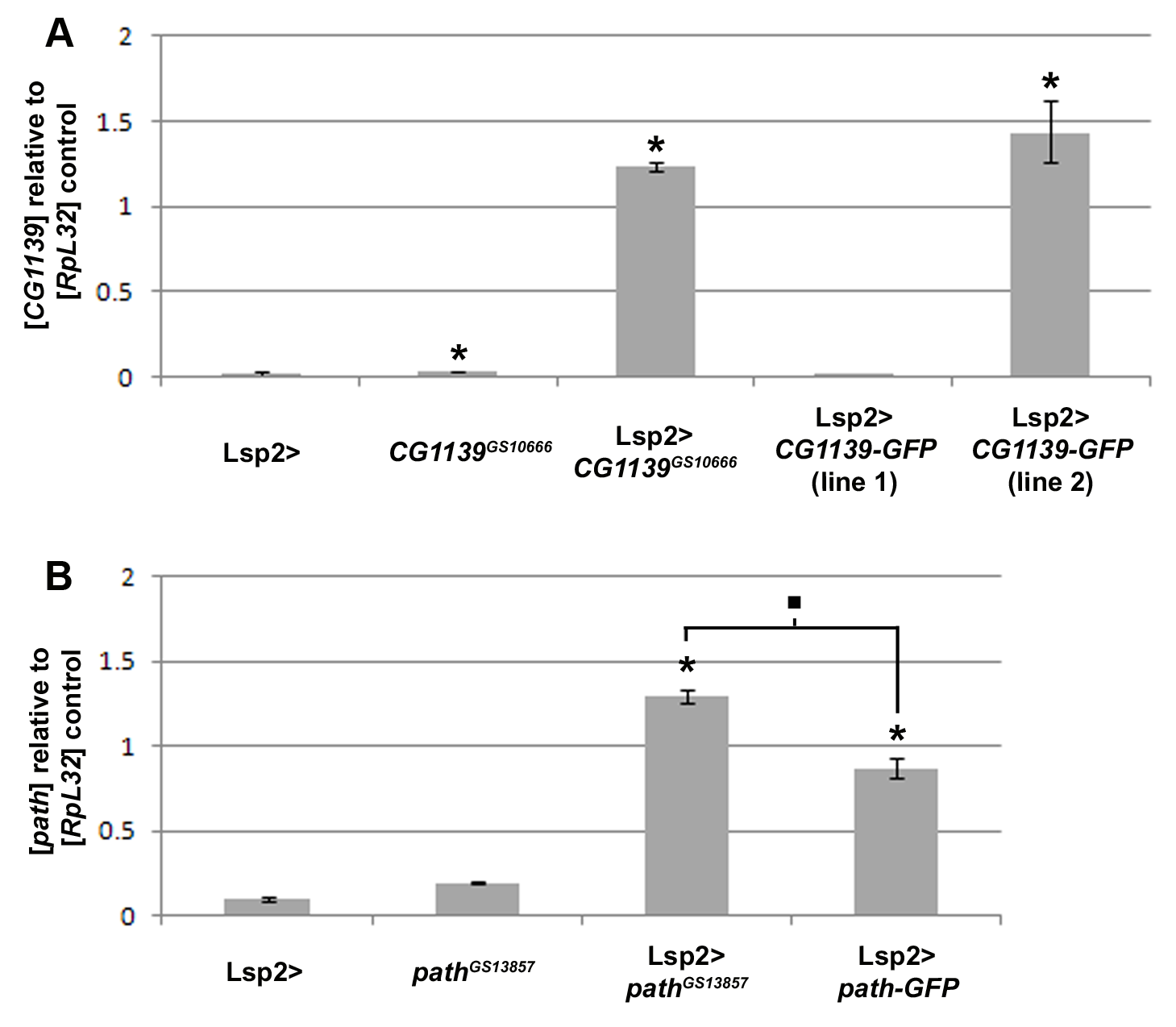

Supplement: Figure S1 — PAT-GFP insertions are expressed at different levels in vivo . Different UAS-PAT-GFP insertion lines and the two Gene Search insertion lines [72] CG1139GS10666 and pathGS13857, were expressed using the Lsp2-GAL4 driver [50], [55], which produces detectable fusion protein in the fat body with all PAT-GFP lines tested (e.g., Figure 6). Levels of CG1139 (A) and path (B) transcripts in fat body RNA preparations were measured using Q-RT-PCR and normalised relative to the levels of the RpL32 housekeeping control transcript. These data revealed a correlation between levels of PAT expression and the growth-promoting activity of specific constructs. The CG1139-GFP line 2 insertion, which gives strong overgrowth and FOXO-dependent cell death phenotypes (Figures 5F, L and R), produced comparable transcript levels to CG1139GS10666, which we have previously employed to overexpress this transporter at high levels (Figure 5C; [49]). path transcripts are normally expressed at moderate levels in the fat body [73]. Levels of path transcripts increased when path-GFP was expressed using the Lsp2-GAL4 driver, but only to about half the level produced by pathGS13857, which gives stronger phenotypes when overexpressed in the eye (Figures 5B, H and N). * (P<0.001) indicates significantly higher levels than Lsp2>control. ■ (P<0.001) indicates significantly lower levels of the fusion transcript than with Lsp2>pathGS1385. (TIF) [file pone.0036616.s001.tif]

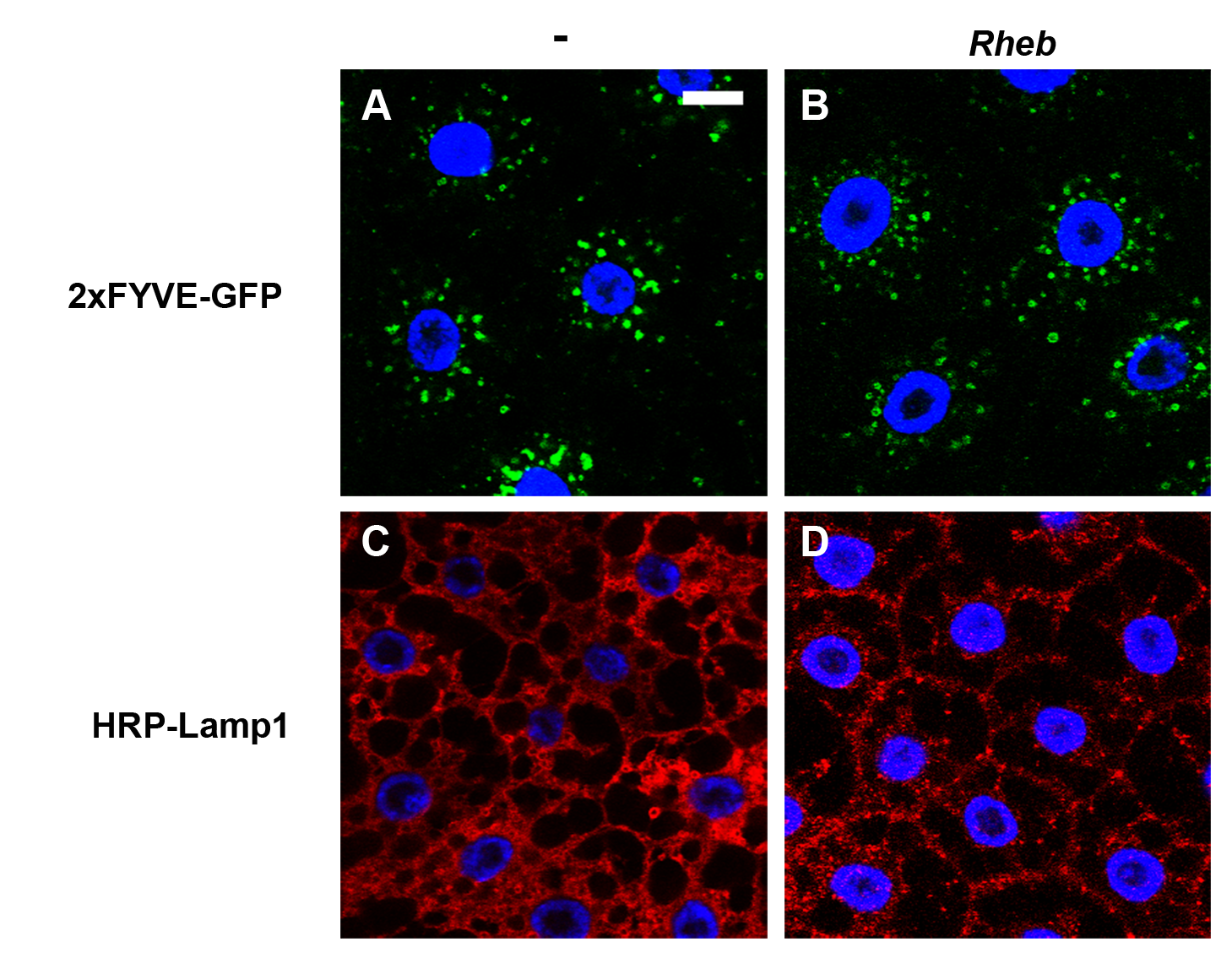

Supplement: Figure S2 — Expression pattern of endosomal markers in the Drosophila larval fat body. (A–D) Figure shows expression of the FYVE-GFP (early endosomal; A, B) and HRP-Lamp1 (late endosomal; C, D) markers in the larval fat body. In the presence (B, D) and absence (A, C) of Rheb, FYVE-GFP (green) is largely confined to a perinuclear region (arrows), while HRP-Lamp1 (red) has a more widespread punctate distribution throughout the cytoplasm. Nuclei are stained with DAPI (blue). Scale bar in A also applies to B and scale bar in C also applies to D; both are 20 µm. (TIF) [file pone.0036616.s002.tif]
