# Supplementary material for: Proton-Assisted Amino Acid Transporter PAT1 Complexes with Rag GTPases and Activates TORC1 on Late Endosomal and Lysosomal Membranes
Source: PLoS One. 2012 May 4;7(5):e36616. doi: 10.1371/journal.pone.0036616 (PMC3344915; doi:10.1371/journal.pone.0036616)
Supplement: Methods S1 — Subcloning, production of transgenic fly lines, measurement of mRNA levels in Drosophila fat bodies, fat body immunostaining. (DOC) [file pone.0036616.s003.doc]

**Methods S1**

***Subcloning***

To make *Flag-PAT1*, the ORF of a full length *PAT1* clone (BX537963) was PCR-amplified using a forward primer with a Flag sequence (DYKDDDDK) inserted directly after the start codon. The PCR product was subcloned into pGEM-T Easy (Promega) and digested out using *Not*I. The fragment was then ligated into a *Not*I digested pcDNA3.1(+) vector and the orientation determined by *Kpn*I digestion prior to sequencing.

To make *path-GFP*, the 3′-end of *path* was PCR-amplified from a putative full-length *path-RA* clone (RH24992) to produce a product with *Bam*HI and *Bgl*II ends that was subcloned into *Bam*HI-digested *pEGFP-N1* (Clontech), such that there was a *Bgl*II/*Bam*HI in-frame fusion at the 3′-end of *path* adjacent to the GFP tag. The complete *path* ORF was then regenerated in the *pEGFP-N1* vector by subsequently subcloning in the 5′ *Eco*RI/*Bam*HI *path* fragment from RH24992.

*CG1139-GFP* was generated by PCR amplification of the 3′-end of a putative full-length clone of CG1139 (LP06969) to produce a product with *Bam*HI and *Bgl*II ends, which was subcloned in frame into *pEGFP-N1* (Clontech). The complete *CG1139* ORF was then regenerated in the pEGFP-N1 vector by subcloning the 5′ *Bgl*II *CG1139* fragment from RH24992 into this construct.

*path-GFP* and *CG1139-GFP* were then excised with *Eco*RI and *Not*I and subcloned into *pUAST* to generate *UAS-path-GFP* and *UAS-CG1139-GFP* respectively. For *Drosophila* S2 cell culture, *path-GFP* was digested with *Eco*RI and *Not*I from *UAS-path-GFP* and then subcloned into *pMT/V5-His B* (Invitrogen).

***Production of transgenic fly lines***

Transgenic lines carrying the *UAS-CG1139-GFP* and *UAS–path-GFP* constructs were generated by standard procedures, microinjecting fly embryos expressing Δ2-3 transposase (*y w*; *Ki pp Δ2-3* flies; kindly provided by Hugo Bellen).

***Measurement of mRNA levels in* Drosophila *fat bodies***

Whole fat bodies were dissected from late third instar larvae in PBS. RNA was extracted (RNeasy Mini Kit, Qiagen) and cDNA synthesized from five fat bodies per sample (High-Capacity cDNA Reverse Transcription Kit, Applied Biosystems). mRNA levels were measured by quantitative real-time PCR using the 7000 Sequence Detection System (Applied Biosystems) and Quantitect® Primer Assays (Qiagen), and normalised against *Ribosomal protein L32* (*RpL32*). Significance values were calculated using Student’s unpaired two-tailed t-test.

***Fat body immunostaining***

Fat bodies from the anterior ends of late third instar larvae were dissected directly in 4% paraformaldehyde in PBS and incubated for 20 min at 4°C prior to washing 3 x 5 min in PBS with 0.3% [v/v] Triton-X (PBST). The tissues were blocked with PBST + 10% [v/v] filter-sterilised goat serum (PBSTG) for 30 min at 20°C, incubated with primary antibody [rabbit anti-HRP (1:4,000; Jackson ImmunoResearch Laboratories, Inc, PA, USA), diluted in PBSTG] at 4°C overnight. The samples were washed 6 x 5 min in PBST, incubated with secondary antibody (Cy5-coupled donkey anti-rabbit; 1:200; Jackson Immuno Research diluted in PBST) for 2 h at 20°C and washed 3 x 5 min in PBST, prior to dissection in PBS and mounting in VectaShield containing DAPI (Vector Laboratories).
